# Unusual Variant of Unicystic Ameloblastoma with CEOT-Like Areas: A Rare Case Report with Review of Literature

**DOI:** 10.1155/2021/2093927

**Published:** 2021-07-17

**Authors:** Venkata Ramanand Oruganti, Shylaja Sanjeevareddygari, Manay Srinivas Munisekhar, Sharath Kumar Reddy Eppalapalli, Raghu Vamshi Vishwakarma, Kiran Kumar Ganji, Kiran R. Halkai, Rahul Halkai

**Affiliations:** ^1^Department of Oral Pathology, SVS Institute of Dental Sciences, Mahabubnagar, Telangana, India; ^2^Oral Pathology Division, Department of Preventive Dentistry, College of Dentistry, Jouf University, Sakaka, Al Jouf, Saudi Arabia; ^3^Periodontics Division, Department of Preventive Dentistry, College of Dentistry, Jouf University, Sakaka, Al Jouf, Saudi Arabia; ^4^Department of Conservative Dentistry and Endodontics, Al-Badar Dental College and Hospital, Kalaburgi, Karnataka, India

## Abstract

Ameloblastoma is an epithelial odontogenic neoplasm with clinical and histological diversity. They are locally invasive tumors with 3 clinical variants such as solid, unicystic, and peripheral ameloblastomas, and the unicystic variant constitutes only 13%. Histologically, it shows diverse microscopic patterns that may occur isolated or in combination with other patterns. The granular cell variant accounts for 3.5% of all ameloblastoma cases. The eosinophilic granules seen in the cytoplasm of the tumor are thought to be lysosomes and presumably contribute to the pathogenesis of the tumor. Although such a phenomenon is rare in unicystic ameloblastoma, granular cell differentiation in solid multicystic ameloblastoma is a well-established phenomenon. In this paper, we present a unique case of unicystic ameloblastoma with granular cell differentiation with a brief review.

## 1. Introduction

Ameloblastoma is a true benign odontogenic tumor of epithelial origin containing enamel organ-like tissue without any hard tissue formation. It was defined by Robinson as “unicentric, nonfunctional, intermittent in growth, anatomically benign and clinically persistent” tumor [1]. It is a locally invasive tumor accounting for 11% among odontogenic tumors in Caucasians [2]. Histologically, plexiform and follicular variants are the two chief patterns, and when certain changes like granular transformation and squamous metaplasia may be noted, they are referred to as granular cell and acanthomatous variants, respectively [3]. The granular cell variant is the least common, but the most aggressive histological type with higher incidence of malignant transformation and distant metastasis [4]. WHO clinically categorized ameloblastomas into solid multicystic, unicystic, desmoplastic, and peripheral ameloblastomas. However, they are similar histologically. Rarely do they present interesting variations microscopically. However, unicystic ameloblastoma (UA) rarely presents with a myriad of histopathological patterns. In this article, an interesting case of UA is presented along with a literature review relevant to its unique microscopic features [5].

## 2. Case Report

**Figure 1:**
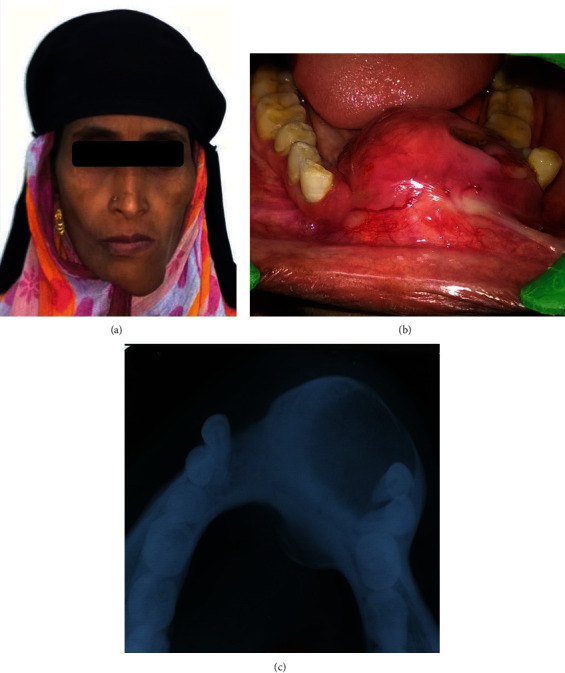
(a) Extraoral swelling; (b) intraoral ulcerated mass with obliteration of labial vestibule; (c) radiographic presentation showing the expansion of cortical plates.

A 45-year-old female patient reported with a swelling in the mandibular anterior region since 4 years. It began as a peanut-sized swelling which progressed to about 6 cm and has increased rapidly during the last two months. Extraoral examination revealed that there was facial asymmetry (Figure 1) with the swelling extending from the right parasymphysis to the left parasymphysis region anterio-posteriorly and superio-inferiorly from the lower lip to the inferior border of the mandible. The skin over the swelling was smooth and was of the same color as the adjacent skin. On intraoral examination, it is approximately 6 × 7 cm in size extending from 43 to 34 obliterating labial and lingual vestibules (Figure 1(b)).

The mandibular left premolars were displaced, and the swelling presented an ulcerated surface on the left side. On palpation, it was hard and nontender. Orthopantamograph (OPG) revealed unilocular radiolucency extending from the mesial aspect of 43 to the mesial aspect of 34, and the occlusal view showed an expansion of labial and lingual plates with intact cortical bone (Figure 1(c)). Sections of the incisional biopsy specimen stained with hematoxylin (H) and eosin (E) revealed odontogenic tumor epithelial cells arranged in sheets, cords, and follicles exhibiting tall columnar peripheral cells with central star-shaped cells resembling the stellate reticulum. The intervening connective tissue was predominantly fibrous. Areas of the lining epithelium were evident made of the parakeratinized stratified squamous epithelium overlying a fibrocellular connective tissue with few chronic inflammatory cells. It was diagnosed as plexiform ameloblastoma followed by surgical removal.

Under all aseptic conditions, GA was administered through naso-endochondral intubation. The patient's face and oral cavity were painted with povidine-iodine and draped. LA with adrenaline 1 : 80,000 was administered as a bilateral inferior alveolar nerve block and mental nerve block. Incision was placed from 46 to 36, and subperiosteal dissection was done bilaterally up to the premolar region both buccally and lingually. Supraperiosteal dissection was done over the anterior mandibular region to expose the tumor. Tumor borders were osteotomized with the help of osteotomes, and the lesion was separated from the mandible. Lingually, genioglossus and geniohyoid muscles were found to be attached to the genial tubercles and were secured. Curettage along with chemical cauterization using Carnoy's solution was done at the lesion site. Hemostasis was achieved. Thorough intraoral irrigation was done. Primary closure was done using 3-0 vicryl with horizontal mattress sutures. Tongue stitch was placed to prevent fall back of the tongue immediately postoperatively and secured extraorally. Ryle's tube was placed, and no intraoral complications were noted. The patient was extubated and shifted to the postoperative ward uneventfully. The healing was uneventful with no recurrence till date.

**Figure 2:**
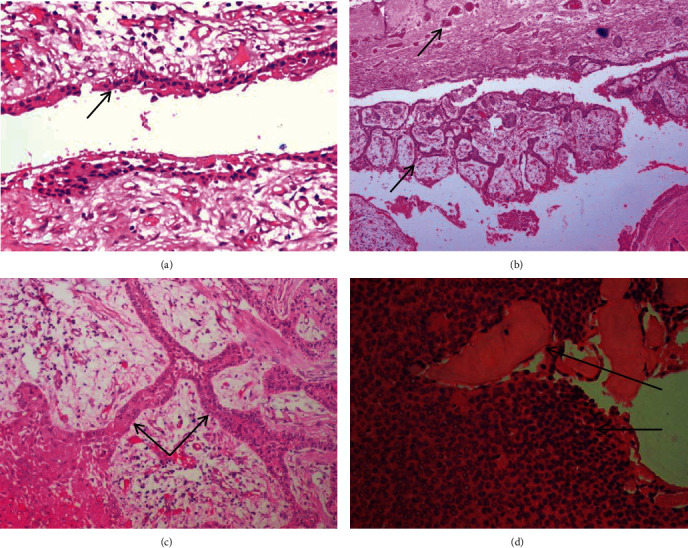
(a) H&E-stained section showing the cystic lumen lined by a thin nonkeratinized stratified squamous epithelium with columnar basal cells at focal areas (×100). (b) H&E-stained section showing epithelial proliferations into both the cystic lumen and the connective tissue wall (×40). (c) H&E-stained section showing odontogenic epithelial cells arranged in interconnecting strands and sheets with granular cells located centrally (×100). (d) H&E-stained section showing darkly stained polygonal cells arranged in sheets with homogenous eosinophilic deposits resembling CEOT-like areas.

The H&E-stained sections of the excisional biopsy specimen revealed a well-defined cystic lumen lined by a nonkeratinized stratified squamous epithelium with basal tall columnar ameloblast-like cells and superficial stellate reticulum-like tissue satisfying Vickers and Gorlin criteria (Figure 2(a)). The epithelial lining showed mural proliferations and proliferations into the cystic lumen in the form of interconnecting strands and cords, with stellate reticulum-like tissue exhibiting granular cell differentiation with eosinophilic granules in the cytoplasm (Figures 2(b) and 2(c)). The intervening connective tissue showed delicate collagen fibers. In some areas, eosinophilic polygonal cells with prominent nuclei and amorphous eosinophilic material resembling calcifying epithelial odontogenic tumor (CEOT) were noticed (Figure 2(d)). A diagnosis of “unicystic granular cell ameloblastoma with calcifying epithelial odontogenic tumor-like areas” was given.

## 3. Discussion

UAs are cystic lesions that present as cysts in their clinical, radiographic, and gross features but histologically have features resembling ameloblastoma-like areas in the lining epithelium of the cystic cavity [6]. Depending upon the histological location of the tumor nodules within the lesional tissue, they have been categorized as intraluminal, luminal, and mural variants [7]. Though it resembles conventional ameloblastoma histologically, it has been separated from it as it differs from it in the following characteristics.

- It is relatively common in younger individuals
- It is more commonly associated with impacted mandibular third molars and hence resembles a dentigerous cyst on a radiograph
- It is less aggressive in its biological behavior with better overall prognosis and decreased recurrence rate [8]

Although granular cell differentiation has been a documented phenomenon in conventional ameloblastomas, such a feature has been infrequently appreciated in UA [9]. According to Broca, they account for 1 to 2% of all jaw tumors and cysts. It is common in the posterior mandibular region (third molar). However, it was noted in the lower anterior region in our case. It is uncommon in fourth and fifth decades with no sex predilection [10]. Ponce et al. reported UA demonstrating histological patterns such as granular cell, basal cell, and acanthomatous patterns and hyaline ring granuloma [11]. However, in our case, granular cell changes and those resembling CEOT were identified. Since such changes are rare (Table 1), the impact of such findings on its biologic behavior is unknown and documentation of few more similar cases may shed light on its pathogenesis and nature [5]. Granular cell ameloblastoma (GCA) is characterized by islands of odontogenic tumor epithelial cells containing peripheral ameloblast-like cells with centrally large oval to polyhedral cells with abundant coarse granules within the cell displacing the nucleus to the periphery of the cells. Electron microscopy revealed that these granules are lysosomes [3]. However, the probable reasons for their occurrence have been speculated as follows:

1. During amelogenesis, in the synthetic and postsecretory stages, ameloblasts usually show an increase in autophagic lysosomes. Similarly, in ameloblastoma, the odontogenic epithelial cells show granular changes due to either lysosomal insufficiency or excessive production of unused materials [9]
2. Aging theory: since granular change was observed in ameloblastomas after two decades of the initial onset of the tumor, it was assumed that aged components get accumulated within the tumor cells and that there is decreased ability of lysosomes to dispose them with increasing age [12]. Thus, increased lysosomes within the tumor cells might indicate enhanced activity to digest the unwanted compounds. However, Neville et al. did not consider it an aging change since it was found in younger patients also [13]
3. Electron microscopy observed apoptotic cell fragments of compact nuclei in granular cells, which were removed by neighboring granular cells, implying that increased tumor cell apoptosis accompanied by phagocytosis could be leading to cytoplasmic granularity [14]
4. However, another electron microscopic study showed that the nuclei of the granular cells were normal without any degenerative changes, and therefore, it was suggested that the presence of numerous lysosomes is indicative of active function and does not represent an aging change [9]

Differential diagnosis for GCA includes granular cell tumor, granular cell myoblastoma, granular cell odontogenic tumor, congenital epulis, and granular cell ameloblastic fibromas. Though the morphology of the granular cells is similar in all these tumors, their histogenesis differs. Secondly, GCA is epithelial in origin and the others are mesenchymal. Immunohistochemistry may help in distinguishing GCA from these tumors [15]. The treatment of ameloblastoma is controversial, and hence, comprehensive history, routine radiographs, proper clinical examination, advanced imaging, and representative biopsy should be considered. It is an established fact that UAs are less aggressive as compared to solid ameloblastomas and are treated successfully with enucleation or curettage [16]. However, since granular cells are associated with aggressive nature with higher incidence of malignancy and metastasis, it would be more appropriate to plan a more radical approach [17].

## 4. Conclusion

In the present case, though incisional biopsy report suggested it to be a plexiform ameloblastoma, enucleation was done as the patient preferred a more conservative approach and its healing was uneventful without any evidence of recurrence till date. Since the excisional biopsy report showed the presence of granular cells and CEOT-like areas, the idea that does it warrant an additional surgery that is more invasive is still questionable. Unless few more such cases are reported, the precise treatment protocols for such lesions cannot be established.

## Figures and Tables

**Table 1:** List of cases reported.

Authors	Year	Cases reported
Abaza et al. [18]	1989	Granular cell odontogenic cyst: a unicystic ameloblastoma with late recurrence as a follicular ameloblastoma
Thillaikarasi et al. [9]	2010	Cystic granular cell ameloblastoma
Ponce et al. [11]	2014	Unusual histological patterns and hyaline ring granulomas in a unicystic ameloblastoma
Motahhary et al. [19]	2014	Granular cell type of a unicystic ameloblastoma: an unusual case and review of the literature
Jain et al. [5]	2017	Unicystic ameloblastoma of the mandible with an unusual diverse histopathology
